# Evaluation of the Effectiveness of an Osseodensification System for Improvement in Bone Density After Delayed Implant Placement: Protocol for a Single-Arm Pilot Study

**DOI:** 10.2196/80200

**Published:** 2026-09-01

**Authors:** Rutuja Karamore, Prasad Dhadse, Ruchita Patil, Sanehi Punse

**Affiliations:** 1 Department of Periodontics and Implantology, Sharad Pawar Dental College and Hospital (SPDC) Datta Meghe Institute of Higher Education and Research (DMIHER), Deemed to be University (DU) Wardha, Maharashtra India

**Keywords:** osseodensification, dental implants, bone density, implant stability quotient, ISQ, Hounsfield units, HU, delayed implant placement, bone-to-implant contact, BIC, resonance frequency analysis, RFA, pilot clinical study, osseointegration

## Abstract

**Background:**

Delayed placement of dental implants often leads to bone resorption, particularly in the posterior region, compromising implant placement. Osseodensification uses specialized rotary instruments to compact and densify bone and may improve bone quality and implant stability. However, its effectiveness in delayed implant placement has not been fully explored.

**Objective:**

This study aims to assess the effectiveness of osseodensification in increasing bone volume and density at regions scheduled for delayed implant placement and to evaluate its effect on implant stability and osseointegration.

**Methods:**

This 9-month single-arm clinical and radiographic study will include 22 systemically healthy patients with D2-D3 bone quality in an edentulous region of the anterior or posterior arch. Preoperative full-mouth ultrasonic scaling, dental hygiene instructions, and cone beam computed tomography (CBCT) imaging will be performed. Bone density will be measured at the coronal (mesial and distal), middle and apical regions of the extraction socket in Hounsfield Units (MU). During implant placement, osseodensification will be used for osteotomy preparation. Implant Stability Quotient (ISQ) values will be assessed using resonance frequency analysis. CBCT imaging will be done at baseline, 3 months, and 9 months post surgery. Recruitment began in July 2025 following ethics approval and trial registration (CTRI/2025/03/082513). The primary outcomes include changes in bone density and implant stability; secondary outcomes assess peri-implant bone healing, osseointegration, and complications.

**Results:**

Recruitment began in July 2025, and as of October 2025, screening and baseline assessments are ongoing. Enrolment of 22 participants was expected by January 2026, with the surgical phase expected to be completed by April 2026. Follow-up evaluations will be performed at baseline, 3 months, and 9 months post surgery. Final data collection and statistical analysis are anticipated by July 2026. No outcome data are yet available because the study is ongoing.

**Conclusions:**

This ongoing pilot study will provide preliminary evidence on the feasibility of osseodensification for delayed implant placement and its effect on bone density and implant stability. The findings may guide methodological refinements and future controlled clinical trials.

**Trial Registration:**

Clinical Trials Registry India CTRI/2025/03/082513; https://tinyurl.com/34562c7b

**International Registered Report Identifier (IRRID):**

PRR1-10.2196/80200

## Introduction

Dental implant placement is a cornerstone procedure in modern dentistry, offering a long-term solution for patients with missing or damaged teeth. Dental implant placement faces challenges such as insufficient bone density, which may require grafting, and risks like infection, poor osseointegration, or mechanical failure [1]. Nevertheless, we must consider that even in healthy individuals and experienced operators, some implant complications (peri-implantitis, bone dehiscence, and impossibility to obtain ideal implant stability) may be a very common situation because of other etiologic agents, such as biomechanical factors or inadequate preparation of the site hosting the implant [2,3]. The presence of systemic diseases (diabetes mellitus, coagulation disorders, and osteoporosis), anticoagulant, bisphosphonate, and aspirin therapy [4], as well as the physiology and anatomy of the treated structure (bone quantity and density and mental nerve not far from the level of the bone crest) could all affect the outcome of the treatment. Other factors depend on the operator (experience, methods, instruments used, and team skills).

Due to decreased bone quality and primary stability, patients with low bone density, such as those with managed osteoporosis, mild to moderate bone loss, especially in the posterior maxilla, older patients, or those receiving long-term steroid therapy (but not high-risk medications like long-term intravenous bisphosphonates), present challenging situations for implant therapy [5,6]. Patients with lower bone density are at increased risk for both primary and secondary implant failure. Primary failure refers to the inability of the implant to integrate correctly with the bone during the healing phase, usually caused by inadequate support from the bone or poor osseointegration. Secondary failure can occur after a period, usually due to complications like peri-implantitis or mechanical overload, which may be more likely in patients with compromised bone density [6-8]. Insufficient bone volume or quality may compromise the initial stability of the implant, increasing the risk of these failures. Such problems necessitate bone-based grafting or sinus lift intervention, but even after these interventions, the risk is still relatively higher for implant failure in such patients with low bone density [9]. The outcome of implant therapy is greatly influenced by bone density, which is frequently categorized as D1, D2, D3, and D4 according to its quality and structure. While D2 bone, which is medium dense and primarily trabecular with a cortical shell, is thought to be the most suitable for implant placement because of its balance of density and vascularity, D1 bone is dense and cortical, providing great primary stability. On the other hand, D4 is the least supportive of the D3 and D4 bones, which are becoming less dense and more porous. Although successful osseointegration requires meticulous expertise, implants put in D2 bone typically have positive results [10].

Different issues may arise when placing implants in D2, where the bone is of medium density, and in D3, where the bone is poor in quality. Dental implants placed in D2 type bone are likely to have enough support, although their placement needs careful attention for good osseointegration. D3 bone, on the other hand, is of lower density and more porous [11]. The primary stability may be poor and the risk of implant failure increases. To counter these difficulties, a bone grafting procedure or a larger or special design of implants may be needed. Implants in D3 bone have a higher risk of complications such as failure of osseointegration or implant loosening, requiring close monitoring during the healing process [12].

Subtractive drilling, which is used in traditional osteotomy preparation for implant placement, removes bone and may further lower density at the implant site, impairing insertion torque and delaying osseointegration [13]. In order to overcome these limitations, osseodensification, first presented by Huwais and Meyer [14] in 2013 was developed as a biomechanical drilling method that preserves and densifies native bone by compacting and autografting bone along the osteotomy walls. Osseodensification increases mechanical stability and improves the bone-to-implant contact (BIC) surface by rotating specifically made burs counterclockwise and without cutting [5]. This method densifies bone along the osteotomy walls by rotating specially made burs counterclockwise [14]. Numerous benefits have been documented by studies, such as enhanced BIC, quicker healing, and better primary stability. It involves the use of specifically designed drills that expand the osteotomy site and compact the surrounding bone, thus preserving it. The key advantages of osseodensification include improved primary stability, especially in cases with lower bone density, and enhanced bone quality around the implant. This technique has shown potential to reduce the need for bone grafting procedures [15,16], promote better osseointegration, and improve overall implant success rates. However, it requires skill and experience to avoid over compaction or potential damage to the bone.

Despite these positive outcomes, the majority of the data is derived from retrospective analyses using diverse methodologies, animal models, or in vitro research. Only a few well-designed prospective clinical trials have evaluated osseodensification for delayed implant placement in moderate-density bone, especially when using stability measures and standardized radiography over an extended period of time [6,8]. Therefore, controlled studies that methodically assess the effects of osseodensification on bone density, primary stability, and osseointegration in vivo are required.

Despite the growing evidence supporting osseodensification, most available studies are retrospective, based on animal or in vitro models, or involve heterogeneous methodologies and follow-up periods. Only a limited number of well-designed prospective studies have evaluated osseodensification specifically for delayed implant placement in moderate-density bone (D2-D3) using standardized radiographic parameters and implant stability measurements over time [6,8]. However, these studies often differ in measurement protocols, timing of evaluations, and implant systems used, making it difficult to compare outcomes or establish clear clinical guidelines.

Therefore, before undertaking large-scale controlled trials, it is necessary to conduct a feasibility-oriented pilot study to assess the practicality, reproducibility, and short-term clinical outcomes of osseodensification under standardized conditions. The present single-arm exploratory study was designed to address this need. By applying uniform cone beam computed tomography (CBCT)–based bone density assessments (in Hounsfield Units [HU]) and resonance frequency analysis (Implant Stability Quotient [ISQ]) at defined intervals, this study aims to generate preliminary clinical data on how osseodensification influences bone density and implant stability in delayed implant sites.

Unlike prior retrospective or ex vivo investigations, this protocol emphasizes methodological standardization and longitudinal follow-up within a real clinical setting. The findings will serve as baseline data for estimating effect sizes, refining study logistics, and informing the design of future randomized controlled trials that can definitively compare osseodensification with conventional osteotomy techniques.

This study will help to bridge the evidence gap and offer recommendations regarding whether osseodensification can be a practical substitute for traditional drilling methods in improving the success of dental implants through clinical and radiographic assessments [17]. Thus, this study aims to assess how well osseodensification works to increase bone density and implant durability in delayed implant implantation within bone quality sites D2 and D3. Changes in peri-implant bone density, insertion torque, and implant stability over time will be evaluated using clinical and radiographic studies.

## Methods

### Overview

This single-arm study will aim at assessing the efficiency of the osseodensification system in enhancing the bone density and volume in cases with delayed implant placement. It will assess the osseodensification impact on the healing of bones and osseointegration, which is by comparing the clinical and radiographic outcomes before surgery and after it.

### Ethical Considerations

This study protocol was reviewed and approved by the Institutional Ethics Committee (IEC), Datta Meghe Institute of Higher Education and Research (deemed to be university [DU]), Wardha, Maharashtra, India (approval number DMIHER(DU)/IEC/2025/561). The study has also been registered with the Clinical Trials Registry of India (CTRI) under the registration number CTRI/2025/03/082513.

Written informed consent will be obtained before any subject is recruited. Through an information sheet and a verbal explanation from the researchers, each participant will receive comprehensive information on the study's goals, methods, potential risks, and anticipated advantages. No personally identifiable information will be revealed in study reports or publications, and confidentiality will be preserved by issuing unique identifying codes. At any point during the study, participants will have the liberty to leave without affecting their ongoing or future treatments.

### Sample Size Calculation

Based on the key outcome variable, implant stability (ISQ values), which will be evaluated at baseline, 3 months, and 9 months post surgery in the same patients, the sample size for this study was estimated. The repeated measures ANOVA framework was chosen because it takes into consideration correlations between several observations made on the same subject.

According to earlier research assessing osseodensification, the average ISQ improvements were between 3 and 5 units, with SDs varying between 1.5 and 2.5. For repeated measures over three time points, a modest effect size (f=0.40) was anticipated using these estimations. The necessary sample size was determined to be 22 patients with α=.05, power=0.90, and correlation among repeated measurements set at 0.5 (G*Power 3.1; Heinrich Heine University Düsseldorf; repeated measures ANOVA, within-subject variables).[18]

This sample size will guarantee feasibility in the framework of an exploratory clinical protocol and offer enough power to identify clinically significant variations in bone density and implant stability over the course of the trial.

The calculation used the formula stated below, where the input parameters are as follows:

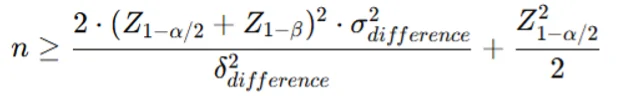



The input parameters are α (for a 95% CI)=1.96, : 0.01 (power=1β=99%)=2.57, and mean difference 4 (SD 1.86).

The minimum paired sample size needed is 22 participants.

### Eligibility Criteria

#### Inclusion Criteria

The inclusion criteria of this study appear to preferentially select patients who are most likely to achieve successful outcomes. Participants are required to demonstrate optimal oral hygiene (plaque score ≤25%), have a thick gingival biotype, intact alveolar bone walls, at least 4 mm of bone at the root apex, and favorable bone quality (D2 or D3). Additionally, an opposing natural tooth and stable adjacent teeth are necessary to ensure proper occlusal function. Patients are restricted to the 30- to 59-year age range. Since it improves soft tissue resilience and promotes superior cosmetic results, a thick gingival biotype is ideal. For adequate structural support, radiographic and clinical assessments must verify that the alveolar bone walls are intact. Additionally, in order to ensure sufficient bone availability, radiography should show at least 4 mm of bone at the root apex. Finally, because D2 or D3 bone quality promotes good osseointegration and implant stability, only these locations will be included.

#### Exclusion Criteria

The study’s exclusion criteria are intended to maximize treatment success, minimize potential problems, and guarantee the selection of qualified patients. To avoid negative reactions and associated difficulties, any individual with known sensitivities to titanium or other implant materials will not be accepted. In addition, patients with systemic illnesses that can impair healing and implant integration, such as diabetes or cardiovascular problems, will be excluded. Due to the higher risk of implant failure, those with active mouth infections or periodontal disease will not be eligible. To prevent any possible hazards to the health of the mother or fetus, women who are currently breastfeeding or pregnant will not be allowed to participate. In addition, due to their impaired healing ability and increased risk of implant failure, people who smoke or have a history of alcohol or drug abuse will not be considered.

This study will be carried out in the Outpatient Department of Periodontology and Implantology, Datta Meghe Institute of Higher Education and Research (DU), Wardha, Maharashtra, India, a teaching hospital for tertiary care dentistry that has a special implant therapy unit. The center was selected because it consistently receives patients requiring implant-supported rehabilitation and has the CBCT imaging and surgical capabilities needed for the study. Recruitment will be done in a methodical way: during routine consultations, consecutive patients who present with edentulous spaces suitable for delayed implant placement will be screened. Sites with D2 or D3 bone quality will be identified by reviewing their dental records and, if available, preliminary radiographs. After confirming basic eligibility by a quick chairside screening of medical history, age, and site features, a calibrated investigator will invite participants who meet these preliminary requirements for a more thorough assessment. Patients who exhibit good dental hygiene (plaque score <25%), intact alveolar walls, and enough bone height will be shortlisted after thorough clinical and radiographic tests, including periodontal charting and CBCT, verifying compliance with the inclusion and exclusion criteria. Before obtaining formal informed permission, the study’s goals, methods, possible dangers, and advantages will be thoroughly presented both orally and in a patient information sheet, giving sufficient time for questions. Participants who are eligible and give their consent will be listed in a recruitment log, given a special identification code, and scheduled for baseline procedures such as preoperative photos, diagnostic castings, and full-mouth ultrasonic scaling. Participants will get instruction on dental hygiene and the significance of attending follow-up appointments at three and nine months, and their contact information (phone number and email) will be recorded to facilitate appointment reminders. Under the principal investigator’s direction, all recruitment efforts will be carried out by qualified investigators following the guidelines of Good Clinical Practice.

### Initial Therapy Procedure

After a thorough examination and diagnosis, full-mouth ultrasonic scaling will be performed as part of the initial therapy. The patients will be given additional plaque control instructions until their plaque scores drop below 25%. Prior to surgery, a diagnostic cast will be constructed for each patient in order to assess the maxillomandibular relationship. Every patient’s preoperative and postoperative clinical photographs will be taken. All patients’ oral hygiene and gingival status will be reviewed on the day of surgery, three, and nine months apart.

### Clinical Measurements

#### Indices

All patients’ oral hygiene and gingival status will be reviewed on the day of surgery, 3, and 9 months apart.

#### Plaque Index

The plaque index (PI) will be calculated by Turesky-Gilmore-Glickman Modification of Quigley-Hein 1970 [19]. The presence of plaque will be evaluated on the labial, buccal, and lingual surfaces of all teeth after the application of the disclosing agent (Table 1).

**Table 1:** Scoring system for the Turesky-Gilmore-Glickman modification of the Quigley-Hein plaque index.

Score	Criteria
0	Absence of plaque.
1	Distinct plaque deposits on the cervical surface of a tooth.
2	Thin, uninterrupted layer of plaque (up to 1 mm) covering the tooth's cervical margin.
3	Plaque band broader than 1 mm but covering less than one third of the crown.
4	Plaque covering not less than one third but less than two thirds of the crown.
5	Plaque that covers two thirds or more of the crown.

The PI value for the tooth will be calculated by splitting all the points around it by two. The PI score for an individual person will be gained by summing the PI scores of all teeth and dividing by the quantity of teeth inspected.

#### Papillary Bleeding Index

A periodontal probe (William Calibrated Probe) will be precisely introduced mesially into the gingival sulcus at the apex of the papilla and will then be advanced coronally to the tip of the papilla. On the same papilla, this was repeated on the distal side. On a scale of 0-4, the degree of potential bleeding was evaluated according to the method described by Checchi et al [20,21].

The papillary bleeding index score per individual will be calculated by adding up all papillary bleeding index scores to the number of surfaces analyzed.

### Radiographic Measurements

Prior to implant insertion, immediately following implantation (0 months) and 3 and 9 months post surgery, bone density (using CBCT) will be measured. Implant stability will be evaluated both immediately following installation (0 months) and 3 and 9 months post surgery. ISQ will be measured using the Osstell device (Osstell AB).

In order to establish the density of the surgical site, a preoperative radiographic evaluation will be performed using CBCT, and the bone density in HU in the coronal (mesial and distal aspect) and apical region of extraction socket, and crestal bone levels will be assessed. The measuring tool in the software will analyze the crestal bone levels at the crest of the bone (first and second threads) and at the apical thirds (last two threads). The bone density will be measured using Planmeca Romexis (Planmeca Oy) software.

### Surgical Procedure

All patients will be given prophylactic antibiotic 1 hour before surgery (amoxicillin 500 mg). Depending on the site of implant placement, a nerve block or infiltration anesthesia will be used to numb the area using a local anesthetic solution of 2% lignocaine solution with 1:100,000 adrenaline.

The dental implant procedure will begin with the mid-crestal incision in the gingiva at the implant site, followed by the gentle reflection of the full-thickness mucoperiosteal flap to expose the underlying bone. Sequential osseodensification drills of increasing diameter will then be used to prepare the bone for implant placement, creating the osteotomy to ensure the site is appropriately sized and shaped for the implant. Osseodensification used during this stage will ensure condensation of native bone and enhance the bone density, facilitating the conditions for improvement in implant stability. After site preparation, a titanium dental implant will be carefully placed into the osteotomy site, ensuring optimal primary stability, which will be crucial for successful osseointegration. The flap will be repositioned and sutured by 3-0 silk suture material, with placement of a healing abutment or cover screw to protect the implant during the healing process.

### Postoperative Care

Patients will be asked to rinse mouth with 0.2 % chlorhexidine (afterwards, twice daily for 2 wk). All patients will be instructed to apply cold fomentation starting immediately and for the next 24 hours. Amoxicillin+clavulanate (625 mg), together with a systemic analgesic aceclofenac+paracetamol+serratiopeptidase (440 mg), will be administered for 5 days. After 7 days, suture removal will be performed.

### Clinical Examination

Implant mobility will be evaluated with the help of the Osstell device instrument (Osstell AB), which is a resonance frequency analysis (RFA) tool. Larger ISQ values will indicate that the implant-bone interface is more stable [22,23].

### Radiographic Measurement

Preoperatively, 3 months, and 9 months post surgery, bone density at the implant site will be evaluated using CBCT. The coronal (mesial and distal) apical portions of the implant site will be assessed for bone density, which will be expressed in HU. To evaluate early changes in bone remodeling, the mean bone density values at baseline and 3 months will be compared. In order to identify additional changes over time and gain insight into the osseointegration and bone healing process, the mean bone density values at 3 and 9 months will also be examined.

### Statistical Analysis

The mean (SD) values will be used to represent all quantitative data. A repeated measures design is preferable to independent or paired 1-tailed *t* tests, which assume just two dependent observations, because measurements will be made for the same participants at three different time points (baseline, three months, and nine months). In order to assess the mean changes in ISQ values and bone density (HU) throughout the three time points, statistical analysis will be carried out using repeated measures ANOVA. Greenhouse-Geisser corrections will be used if the sphericity assumption is violated. To find significant differences between particular time points (eg, baseline vs 3 months, baseline vs 9 months, and 3 months vs 9 months), post hoc pairwise comparisons with Bonferroni adjustment will be performed. The significance level will be set at *P*<.05.

Descriptive statistics (frequency and percentage) will be presented for categorical variables (eg, implant survival and presence or absence of peri-implant disease).

### Rationale for the Study’s Design

As a substitute for being a randomized controlled trial (RCT), this trial is intended to be a single-arm clinical research. This decision was made for two reasons:

1. Phase of exploration and proof-concept: Assessing the viability and efficacy of osseodensification in enhancing bone density and implant stability in delayed implant placement, especially in locations with D2 and D3 bone quality, is the main goal of this protocol. Before evaluating more extensive comparison studies, a single-arm design enables targeted evaluation of changes over time within the same group of patients, given the paucity of prospective clinical evidence in this field.
2. Ethical and practical considerations: Osseodensification is anticipated to be advantageous for all patients who are enrolled in this trial because they all have poor bone conditions. Participants in a control group undergoing traditional osteotomy may be at increased risk of experiencing inadequate implant stability or failure if this procedure is not used. Consequently, a within-subject repeated measures design was judged to be both ethically and therapeutically appropriate for this exploratory study.

### Primary and Secondary Outcome Variables

This study’s primary outcome variables will involve evaluating bone density and volume changes in delayed implant placement locations using radiographic techniques including CBCT to ascertain how the osseodensification system affects compaction and bone preservation. RFA will be used to determine the ISQ, which is another important component. Additionally, the study will use radiographic techniques to analyze BIC over time in order to evaluate osseointegration success.

The clinical and radiographic evaluation of bone healing, as determined by peri-implant bone level changes using CBCT, will be the main emphasis of the secondary outcome variables. Functional loading results, the absence of implant mobility, and the absence of peri-implant radiolucency will also be used to assess the implant success rate. In order to examine the long-term efficacy and safety of implants placed using the osseodensification system, the study will also monitor complication rates, such as early or late implant failure, marginal bone loss, and the incidence of peri-implantitis.

## Results

### Overview

Since this is a study protocol, no analyzed data are currently available. Recruitment for this pilot single-arm study began in July 2025, following institutional ethics approval and trial registration (CTRI/2025/03/082513). As of October 2025, participant screening and baseline assessments were ongoing. The target sample (N=22) is expected to be fully enrolled by January 2026.

The surgical phase, involving delayed implant placement using the osseodensification technique, is scheduled for completion by month 6 (April 2026). Clinical and radiographic follow-up evaluations, including CBCT-based bone density and implant stability (ISQ) measurements, will be conducted at baseline, 3 months, and 9 months post surgery.

Final data collection and statistical analysis are anticipated by month 12 (July 2026). The study is ongoing, and no outcome data are yet available (Table 2).

**Table 2:** Study timeline outlining recruitment, surgical phase, and follow-up assessments.

Study activity or phase	Timeline	Details
Recruitment start	July 2025	Recruitment initiated; screening and enrollment of eligible participants began
Current status	October 2025	Recruitment and baseline assessments are underway
Expected completion of enrolment	January 2026	Target sample size (N=22) expected to be fully recruited
Surgical phase	April 2026 (month 6)	Completion of delayed implant placement using the OD^a^ technique
Follow-up evaluations	Baseline, 3 months, and 9 months post surgery	Clinical and radiographic assessments for bone density and implant stability (ISQ^b^ and CBCT^c^)
Final data collection and analysis	July 2026 (month 12)	Completion of all follow-ups and commencement of statistical analysis

^a^OD: osseodensification.

^b^ISQ: Implant Stability Quotient.

^c^CBCT: cone beam computed tomography.

### Expected Outcomes

It is hypothesized that osseodensification may enhance mechanical retention and primary implant stability by increasing peri-implant bone density and bone-to-implant contact. Within the scope of this pilot study, osseodensification is expected to demonstrate potential improvements in bone compaction and implant stability when compared to baseline values in D2-D3 bone sites.

Based on previous preclinical and limited clinical evidence, it is anticipated that osseodensification could contribute to improved early osseointegration and reduced micromotion. However, these outcomes remain to be verified, as this exploratory protocol is designed primarily to assess feasibility, short-term trends, and methodological reliability, rather than to establish definitive clinical efficacy.

The findings from this pilot study will therefore serve as preliminary data for refining hypotheses, estimating effect sizes, and informing the design of future RCTs comparing osseodensification with conventional osteotomy techniques.

## Discussion

### Principal Findings

The purpose of this study was to assess how osseodensification impacted implant stability and bone density following delayed implant implantation, specifically in locations with D2 and D3 bone quality. It is predicted that, in contrast to what has previously been seen with traditional osteotomy procedures, osseodensification will improve peri-implant bone compaction, raise insertion torque and RFA values, and help to produce more predictable osseointegration.

According to previous research, osseodensification may accelerate osseointegration, improve BIC, and maintain trabecular bone. For instance, osseodensification considerably raised primary stability and peri-implant bone density as compared to the baseline [24]. In preclinical animal models, it was shown that osseodensification leads to more compact peri-implant bone and enhanced secondary stability [13,17]. Comparing osseodensification to traditional drilling, retrospective clinical research [25] and systematic reviews [6,16] have also shown that osseodensification results in less marginal bone loss and fewer complications. Despite these promising findings, the majority of earlier research was restricted to retrospective analysis, ex vivo studies, or animal trials. In order to fill this gap, this study will provide prospective clinical data under controlled circumstances.

Achieving adequate primary stability in individuals with low bone density, especially those with D3 or D4 bone, is difficult and raises the risk of both early and late implant failures [26]. In order to prepare for an osteotomy, the traditional drilling method removes the bone, which could further reduce bone density and delay osseointegration and healing [27]. Huwais and Meyer [14] established the relatively new process of osseodensification in 2013, which compacts and autografts the bone along the osteotomy walls rather than removing it. Specially manufactured burs that rotate counterclockwise at regulated pressures and speeds are used to achieve this [5]. According to one comparative study [24], osseodensification enhances the initial mechanical stability of the implant, maintains bone mass, and increases peri-implant bone density.

Research contrasting osseodensification with traditional drilling has yielded encouraging results [28] and showed that implants inserted with osseodensification had more BIC and higher insertion torque than implants inserted using conventional drills. Similarly, even in low-density bone sites found that osseodensification improved implant stability in the posterior maxilla [15]. It was further shown in an animal investigation that osseodensification resulted in improved peri-implant bone compaction and more consistent bone growth along the osteotomy walls [13].

In addition, osseodensification has been shown to be helpful in enabling sinus floor elevation and ridge extension treatments without requiring extra grafting materials. Also, osseodensification has shown better results in sinus lift procedures because of the increased bone density and reported successful ridge expansion with osseodensification in narrow alveolar ridges [29].

Even if there is increasing evidence for osseodensification, some systematic reviews have stressed the necessity for larger, carefully planned randomized clinical studies to confirm these results [6]. In addition, to attain the stated benefits, operator expertise and accurate technique application are essential [30].

Also, osseodensification may reduce complications, according to clinical observations. According to a retrospective investigation [25], sites treated with osseodensification had lower rates of marginal bone loss and implant movement. Furthermore, osseodensification maintains the bone’s vascularity, which may lower the likelihood of peri-implantitis and early failures [16].

Osseodensification seems to reduce the amount of time needed for osseointegration in the healing process. Around implants inserted with osseodensification, a histologic investigation [31] showed enhanced new bone production and quicker bone remodeling. Furthermore, implants implanted with osseodensification showed less micromotion and improved secondary stability [17], which could help ensure long-term success.

This protocol’s prospective design, which includes systematic follow-up at baseline, 3, and 9 months, is one of its main strengths. It allows for the study of both early and intermediate changes in implant stability and bone density. Objective and repeatable outcome metrics are provided by the use of RFA for implant stability and CBCT for radiographic evaluation.

One of the retrospective cohort study [32] evaluated 163 patients (329 implants) treated in the posterior maxilla between 2005 and 2011, comparing implants placed in native bone, direct sinus lift with simultaneous implant placement, and direct sinus lift with delayed implant placement over a minimum follow-up of 5 years (mean 7.0, SD 1.9 years). Radiological parameters, including bone crest level, bone loss, and vertical bone gain, as well as implant success and survival, were analyzed. Bone loss and implant outcomes were similar across all groups, with no significant differences. Graft resorption occurred primarily during the first 12 months but stabilized thereafter, remaining consistent through 5 years of follow-up. Overall, implants placed in native bone or following sinus lift (simultaneous or delayed) showed comparable long-term success and survival, with early graft height reduction followed by stabilization [32]. In another retrospective study [33], less traumatic magnetoelectric versus conventional single-tooth extraction in premolar and molar sites were compared to evaluate alveolar ridge volume preservation. Forty-eight patients were allocated to the test group (20 sites, less traumatic extraction with tooth sectioning and magnetoelectric root subluxation) or control group (28 sites, conventional extraction). Alveolar socket contours were captured intraoperatively and at four months using a laser intraoral scanner, converted to Digital Imaging and Communications in Medicine format, and analyzed volumetrically and for surface area. Significant reductions in anatomical features were observed in all groups (*P*<.001), with mean final ridge volumes of 0.87 (SD 0.34) cm³ for less traumatic extractions and 0.66 (SD 0.19) cm³ for conventional extractions. In molar sites, volume loss was significantly lower with less traumatic extraction (Δ volume −0.30, SD 0.10 cm³, Δ volume % −22.3, SD 8.4%) versus conventional extraction (Δ volume −0.59, SD 0.10 cm³, Δ volume % −44.3, SD 5.8%; *P*<.001). Overall, less traumatic extraction better preserved alveolar crest volume, particularly in molar sites [33]. Another study evaluated the navigated antral bone expansion technique for implant placement in the atrophic posterior maxilla. Thirty-seven partially edentulous patients with 4-7 mm of residual bone were treated using navigated antral bone expansion to increase bone height. Pre- and postsurgical distances between the alveolar ridge and sinus floor were compared using paired-samples *t* tests, and angular deviations between planned and actual implant trajectories, as well as postsurgical complications, were assessed. Data from 35 patients showed a significant mean bone height increase of 3.96 (95% CI 3.62-4.30; *P*<.005) mm, with no postoperative complications observed. The mean angular deviation ranged from 12.7° to 34.9° (mean 25.17°, SD 5.10°). These findings suggest that the navigated antral bone expansion technique provides a significant and minimally invasive bone augmentation option for managing the atrophic posterior maxilla [34].

Nonetheless, it is necessary to recognize some limitations. Since improvements will only be evaluated in relation to baseline values, the single-arm design without a comparator group (conventional osteotomy) limits the capacity to draw concrete conclusions about the superiority of osseodensification. The 9-month follow-up period might not accurately represent long-term implant survival and performance, even though it is long enough to record short- to midterm outcomes of implant stability and bone density. Given the technical sensitivity of osseodensification, operator-related procedure variability may also affect results, even with calibration efforts. In addition, while the inclusion criteria, which concentrate on individuals with intact alveolar walls, adequate oral hygiene, and D2-D3 bone quality, lessen confounding, they may also introduce selection bias and restrict external validity to more diverse real-world populations.

In both high- and low-density bone sites, the findings of this exploratory study will be used as pilot data to guide the design of RCTs that compare osseodensification with traditional drilling. The impact of baseline patient characteristics, including bone quality, systemic diseases, oral hygiene level, or site location on the efficacy of osseodensification should also be investigated in larger research. Furthermore, to demonstrate the external validity and practicality of this method, practical clinical trials with bigger and more varied patient groups would be required.

Presentations at national and international dental research conferences and the submission of full-length publications to peer-reviewed journals will be used to share the trial’s findings. To help with knowledge translation and incorporation into clinical practice, the results will also be shared with the institution’s participating patients and doctors.

It is anticipated that the trial will demonstrate, within the constraints of this single-arm exploratory protocol, how osseodensification may improve bone density and implant durability in delayed implant implantation at locations with D2 and D3 bone quality. The expected results will assist in establishing feasibility, produce effect size estimates, and guide the design of future RCTs that can more definitively evaluate the role of osseodensification in implant dentistry, even though definitive conclusions cannot yet be formed.

### Conclusions

The osseodensification approach exhibits encouraging potential to improve bone density, implant stability, and early osseointegration in delayed implant placement, especially in D2-D3 bone sites, within the constraints of this current single-arm pilot trial. Osseodensification may be able to get around the drawbacks of traditional osteotomy by conserving and compacting native bone instead of removing it, creating a more advantageous biomechanical environment for implant integration and long-term success.

The standardized clinical and radiographic technique used in this trial will produce important preliminary evidence regarding the feasibility and biological benefits of osseodensification, albeit conclusive outcome data are still lacking. The findings will help determine evidence-based clinical guidelines for maximizing implant success in impaired bone conditions, assist in the establishment of more substantial RCTs, and promote the improvement of future protocols.

## Abbreviations

BIC: bone-to-implant contact
CBCT: cone beam computed tomography
CTRI: Clinical Trials Registry of India
DU: deemed to be university
HU: Hounsfield Units
IEC: Institutional Ethics Committee
ISQ: Implant Stability Quotient
PI: plaque index
RCT: randomized controlled trial
RFA: resonance frequency analysis
